# Effectiveness of Virtual Reality Training in Improving Outcomes for Dialysis Patients: Systematic Review and Meta-Analysis

**DOI:** 10.2196/58384

**Published:** 2025-01-08

**Authors:** Xin Kang, Yiping Zhang, Chaonan Sun, Jiaxin Zhang, Zhe Che, Jinhui Zang, Rongzhi Zhang

**Affiliations:** 1 Department of Center for Hemodialysis The Second Hospital of Dalian Medical University Dalian China

**Keywords:** virtual reality, VR, training, dialysis, hemodialysis, peritoneal dialysis, chronic kidney disease, rehabilitation, quality of life, meta-analysis

## Abstract

**Background:**

Virtual reality (VR) training uses computer-generated simulations that enable users to engage with immersive virtual environments, simulating real-world activities or therapeutic exercises. This technology is increasingly recognized as a promising intervention to address the physical and psychological challenges faced by dialysis patients, who frequently experience diminished physical function, social isolation, and emotional distress associated with prolonged treatment regimens. Given the increasing prevalence of dialysis patients and the limitations of conventional rehabilitation approaches, VR presents a novel, interactive method that has the potential to enhance patient well-being and improve quality of life.

**Objective:**

This meta-analysis aimed to evaluate the effectiveness of VR training interventions for dialysis patients, with a focus on assessing their impact on motor abilities, psychological symptoms (specifically anxiety and depression), social functioning, and self-efficacy. This analysis also explores whether VR can offer comprehensive benefits to support both the physical and mental health of dialysis patients.

**Methods:**

The meta-analysis was conducted following Cochrane guidelines. Comprehensive searches were performed across major databases, including China National Knowledge Infrastructure, Wanfang database, China Science and Technology Journal Database, China Biomedical Literature database, Cochrane library, Web of Science, PubMed, and Embase, encompassing all studies up to December 2023. Inclusion criteria targeted studies assessing VR’s impact on motor performance, psychological well-being, social functioning, and self-efficacy in dialysis patients. Two reviewers independently extracted data and assessed methodological quality using Cochrane’s risk of bias criteria, ensuring data synthesis reliability.

**Results:**

A total of 12 studies, involving 625 dialysis patients in total, met the inclusion criteria. The meta-analysis demonstrated that VR training led to significant improvements across multiple domains. VR interventions were associated with improved physical capacity, evidenced by higher scores in the 6-minute walk test (standardized mean difference [SD]=29.36, 95% CI 14.32-44.4, *P*<.001, *I*^2^=46%). VR training was associated with significant reductions in depression (SD=–6.30, 95% CI –7.14 to –5.47, *P*<.001, *I*^2^=96%) and anxiety (SD=–8.91, 95% CI –9.69 to –8.14, *P*<.001, *I*^2^=95%). In addition, VR interventions enhanced social functioning (SD=16.20, 95% CI 14.49-17.9, *P*<.001, *I*^2^=72%), and improved self-efficacy (SD=20.47, 95% CI 18.55-22.39, *P*<.001, *I*^2^=99%). However, VR training did not yield significant differences in gait speed, balance, or functional tests (Ten Sit-to-Stand Test, Five Sit-to-Stand Test, Sixty Sit-to-Stand Test, Timed Up and Go Test, and fatigue) compared with control groups.

**Conclusions:**

The findings suggest that VR training is a promising intervention for dialysis patients, providing benefits in physical endurance, social engagement, and psychological well-being. Despite these advantages, VR remains underused among peritoneal dialysis patients compared with hemodialysis patients. Further studies with larger sample sizes and more refined experimental designs are recommended to validate these results and support VR as a complementary tool in the holistic care of dialysis patients.

## Introduction

End-stage renal disease (ESRD) signifies a severe impairment in kidney function, rendering the body incapable of meeting normal metabolic demands. This condition is chronic and progressive, with a global prevalence reaching 442.13 per million [1]. Dialysis, encompassing both hemodialysis (HD) and peritoneal dialysis (PD), serves as a common therapeutic approach for ESRD, substituting renal filtration function albeit necessitating frequent hospital visits, significantly impacting patients’ lives. Prolonged dialysis treatment often precipitates complications such as anemia, muscle wasting, skeletal disorders, and cardiovascular ailments. Exercise therapy plays a pivotal role in managing patients with ESRD by yielding multifaceted benefits, including enhancements in cardiovascular function [2], alleviation of fatigue and sleep disturbances, bolstering mental health by mitigating anxiety and depression [3], regulating blood pressure, managing anemia [4], and optimizing dialysis adequacy [5]. Furthermore, regular exercise effectively retards the deterioration of muscle strength [6] and maintains superior physical function. Despite extensive research corroborating the merits of exercise in augmenting hemodialysis patients’ quality of life, there remains a notable deficiency in exercise awareness, knowledge application, and adherence among patients with ESRD [7] predominantly attributable to inadequate comprehension, deficient self-management skills, and low compliance levels.

Virtual reality (VR) technology represents a computer-generated simulation environment enabling user interaction within virtual realms, engendering an immersive experience that transports individuals into diverse environments [8]. Within the medical sphere, VR has gained traction and is increasingly adopted. VR facilitates immersive encounters that divert patients’ attention from the discomfort and anxiety associated with medical procedures, such as dialysis treatment [9]. By creating immersive and interactive virtual environments, VR offers a unique platform to improve operational skills, health knowledge, and psychological resilience among these patients.

The application of VR in dialysis begins with its role in enhancing operational skills and ensuring treatment safety. Patients can engage in simulated environments that replicate dialysis machine operations and scenarios. This allows them to practice procedures and familiarize themselves with equipment without the inherent risks associated with real-world settings. Such training not only reduces procedural errors but also boosts confidence and competence in managing treatments, ultimately contributing to better treatment adherence and patient safety.

In addition, VR serves as a powerful educational platform in dialysis care. It facilitates the delivery of comprehensive health education, covering critical aspects such as medication management, dietary guidelines, and health monitoring techniques [10]. By engaging patients in interactive simulations, VR enables them to acquire a deeper understanding of their treatment regimen and develop essential self-management skills. This educational empowerment is pivotal in encouraging patients to take an active role in their health management, thereby improving treatment outcomes and enhancing overall quality of life.

Furthermore, VR addresses the significant psychological challenges faced by dialysis patients. Long-term treatment often leads to emotional stress and psychological burdens, including conditions such as anxiety and depression. VR environments provide a therapeutic avenue for relaxation and emotional support. Patients can participate in virtual mindfulness exercises, guided relaxation sessions, or social interactions within a controlled setting. These activities help alleviate stress, improve mood, and foster emotional resilience among patients, thereby enhancing their psychological well-being.

VR training offers significant advantages over traditional physical therapy for treating dialysis patients. First, VR technology provides highly immersive experiences that enhance patient engagement and motivation. Dialysis patients often face long-term treatments and physical limitations, and VR environments immerse them in compelling experiences that encourage greater participation in rehabilitation training. Second, VR allows for personalized treatment plans tailored to individual patient needs, including adjusting training difficulty and environment to accommodate varying abilities and therapeutic requirements. This personalized approach enhances treatment efficacy and specificity.

Furthermore, VR training provides a safer therapeutic environment with better control mechanisms. Conducting exercises and rehabilitation in virtual environments reduces real-world safety risks for patients, while health care teams can monitor progress and responses in real time to adjust treatment plans promptly. In addition, VR systems can collect detailed data such as movement skills and physiological responses, offering crucial insights for health care professionals to optimize treatment plans and deliver personalized care.

Conducting a meta-analysis on the application of VR in dialysis patients holds significant clinical implications. Systematically synthesizing data from multiple studies allow for a comprehensive evaluation of VR therapy’s actual impact on improving quality of life and enhancing rehabilitation outcomes in dialysis patients. Such meta-analysis not only compares VR training with traditional physical therapy but also identifies potential biases and inconsistencies in research, providing scientific grounds for designing future clinical trials and refining treatment strategies. Therefore, exploring the effectiveness of VR through meta-analysis in dialysis patients not only deepens understanding of its clinical role but also informs future clinical decisions and rehabilitation strategies based on robust scientific evidence.

## Methods

### Overview

This meta-analysis was conducted in accordance with the PRISMA (Preferred Reporting Items for Systematic Reviews and Meta-Analyses) guidelines. The PRISMA checklist is provided in Multimedia Appendix 1.

### Search Strategy

We conducted a comprehensive literature search across 8 databases, namely China National Knowledge Infrastructure (CNKI), Wanfang database, China Science and Technology Journal Database, China Biomedical Literature database (SinoMed), Cochrane library, Web of Science, PubMed, and Embase. We used a combination of subject terms and free terms tailored to each database. The search was performed for studies published from inception up to December 2023. Two researchers independently executed the searches and managed the retrieved literature using EndNote software. Detailed search strategies for each database are provided in Multimedia Appendix 2.

### Inclusion and Exclusion Criteria

Textbox 1 shows the inclusion and exclusion criteria.

**Inclusion and exclusion criteria.**

**Inclusion criteria**

- Study design: randomized controlled trials following the Population, Intervention, Comparison, Outcome framework.
- Population: adults aged ≥18 years undergoing dialysis (hemodialysis or peritoneal dialysis).
- Intervention: exercise training incorporating virtual reality.
- Comparison: conventional hemodialysis care interventions (including health education and routine exercise) or exercise not based on virtual reality.
- Outcome measures: motor ability, psychological symptoms, social functioning, and self-efficacy.
- Language: studies published in Chinese or English.

**Exclusion criteria**

- Duplicate publications.
- Non-Chinese or non-English literature.
- Studies with incomplete data (eg, missing outcome indicators).
- Conference abstracts and review articles.

### Data Extraction

Two researchers independently extracted data from the included studies, including details such as author, publication year, country, sample size, intervention specifics, and outcome measures. Studies were initially screened based on titles and abstracts, followed by a full-text review to ensure they met the inclusion criteria. Discrepancies between the 2 researchers were resolved through discussion, and if consensus could not be reached, a third researcher was consulted for final arbitration.

To ensure comprehensive data extraction, we contacted study authors by email to request missing or unclear data. Studies with unresolved data issues were excluded from the meta-analysis.

### Quality Assessment of Literature

The quality of included studies was assessed using the Cochrane Collaboration’s risk of bias tool. We evaluated studies on several criteria, such as random sequence generation, allocation concealment, blinding of participants and personnel, blinding of outcome assessment, incomplete outcome data, selective reporting, and other potential biases. Each criterion was classified as low risk, unclear risk, or high risk of bias [11].

### Statistical Methods

Statistical analyses were performed using RevMan (version 5.4) software and State (version 17) provided by the Cochrane Collaboration. We analyzed both continuous and dichotomous variables, reporting relative risk with 95% CIs. A fixed-effects model was used for the meta-analysis of outcome indicators. We assessed heterogeneity among studies using the chi-square test and *I*-squared (*I*^2^) statistic, with *I*^2^ values of 25%, 50%, and 75% indicating low, moderate, and high levels of heterogeneity, respectively. Publication bias was evaluated through funnel plots and Egger regression test [12]. A 2-tailed *P* value of ≤.05 was considered statistically significant [13]. Due to the limited number of studies, further subgroup and sensitivity analyses were not performed.

In cases where data were incomplete or unclear, we sought additional information from the authors. Studies for which relevant data could not be obtained were excluded from the systematic review and meta-analysis.

## Results

### Results of Literature Search

**Figure 1:**
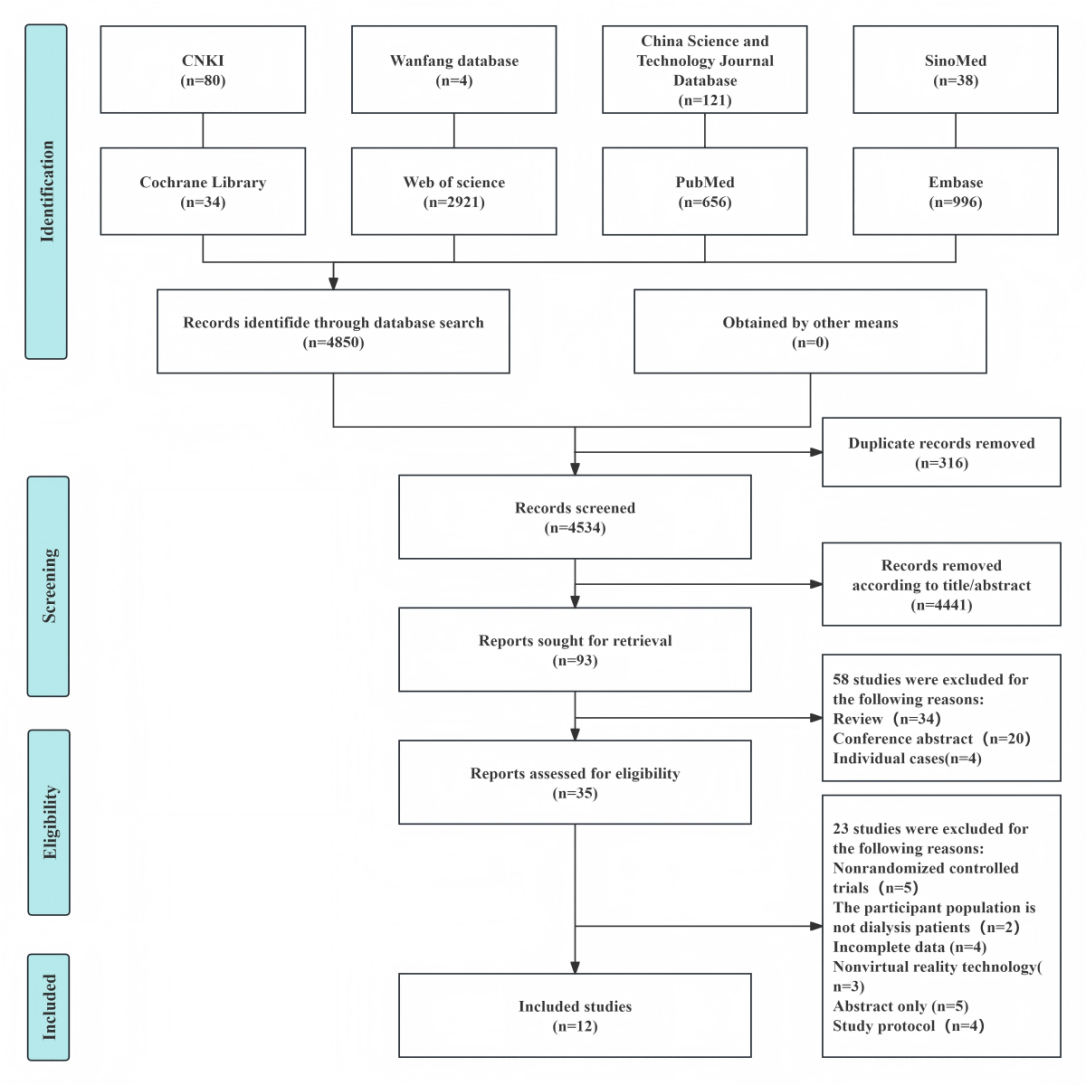
Flowchart of the study selection process.

A total of 4850 articles were initially retrieved from the literature search, comprising 80 from CNKI, 4 from Wanfang database, 121 from China Science and Technology Journal Database, 38 from SinoMed, 34 from the Cochrane library, 2921 from Web of Science, 656 from PubMed, and 996 from Embase. After a systematic screening process, 12 articles were ultimately included. The detailed screening process and the reasons for exclusion are illustrated in Figure 1.

### Basic Characteristics of Included Studies

The included studies were conducted across 7 countries: namely, Poland [14], Spain [15,16], China [17-21], Qatar [22], South Korea [23], Germany [24], and Brazil [25]. One study used a 3-arm design [24], while the remaining 11 studies used a 2-arm design. Of the 12 trials, 2 focused on patients undergoing PD, while the remaining 10 involved patients undergoing HD [17,21]. Five trials compared VR training with conventional care [18-21,23], 2 trials included specified interventions in the control group [14,16], and the remaining trials compared VR training with other types of interventions, such as printed materials [17], exercise programs based on daily habits [15,18], and nurse-led exercise programs [16,22]. Summary of included studies are provided in Multimedia Appendix 3. Details regarding the number of participants, interventions, and outcomes are presented in Table 1. Further information on the interventions under experimental and control conditions is detailed in Multimedia Appendix 2.

**Table 1:** Characteristics of the 12 included studies.

Study	Country	Type	Number (n)				Duration		Time of data collection		Outcomes
			Total	Experimental group	Control group						
Turoń-Skrzypińska et al [14]	Poland	HD^a^	85	39	46	1 year		At the time of entering the research project and following month 3		Depression and anxiety	
Lee et al [17]	China	PD^b^	23	12	11	8 times		At baseline (pretreatment), within the first week after recruitment, and at posttreatment		Knowledge proficiency, procedure competence, and self-efficacy	
Segur‐Ortí et al [15]	Spain	HD^a^	18	9	9	20 weeks		At baseline, after 16 weeks of intradialysis combined exercise, and by the end of four additional weeks of exercise		STS-10^c^, STS-60^d^, gait speed, one-leg heel-rise tests, and 6MWT^e^	
Martínez-Olmos et al [16]	Spain	HD^a^	56	28	28	12 weeks		At baseline, at 12 weeks, and at 24 weeks		The 4-m gait speed test, SPPB^f^, TUG^g^ test, OLST^h^ for balance, STS-10^c^, STS-60^d^, 6MWT^e^, and adherence to the exercise program	
Lei et al [18]	China	HD^a^	42	21	21	6 mouths		Before enrollment, at 3 months, and 6 months after enrollment		Self-efficacy for exercise, risk of falls, and quality of life	
Zhou et al [22]	Qatar	HD^a^	73	37	36	4 weeks		At baseline and at 4 weeks		Depression and user experience	
Chou et al [19]	China	HD^a^	64	32	32	4 weeks		Before intervention and after 4 weeks of intervention		Fatigue, BUN^i^, creatinine, albumin, and hemoglobin	
Cho and Sohng [23]	South Korea	HD^a^	46	23	23	8 weeks		At baseline and after intervention		Physical fitness, body composition, and fatigue	
Schinner et al [24]	Germany	HD^a^	32	12+9	11	12 weeks		—^j^		Functional capacity, serum biochemistry, muscle strength, muscle circumference, and body composition	
Maynard et al [25]	Brazil	HD^a^	40	20	20	12 weeks		After intervention		Functional capacity, quality of life, and depressive symptoms	
Xuelian et al [20]	China	HD^a^	70	35	35	—^j^		Before intervention and after intervention		Anxiety, depression, quality of life, and treatment compliance	
Lina and Ke [21]	China	PD^b^	76	38	38	3 months		—^j^		Activity level and 6MWT^e^	

^a^HD: hemodialysis.

^b^PD: peritoneal dialysis.

^c^STS-10: sit-to-stand 10.

^d^STS-60: sit-to-stand 60.

^e^6MWT: 6-min walking test.

^f^SPPB: short physical performance battery.

^g^TUG: timed up-and-go.

^h^OLST: one-legged stance test.

^i^BUN: blood urea nitrogen.

^j^Not applicable.

### Risk of Bias Assessment

**Figure 2:**
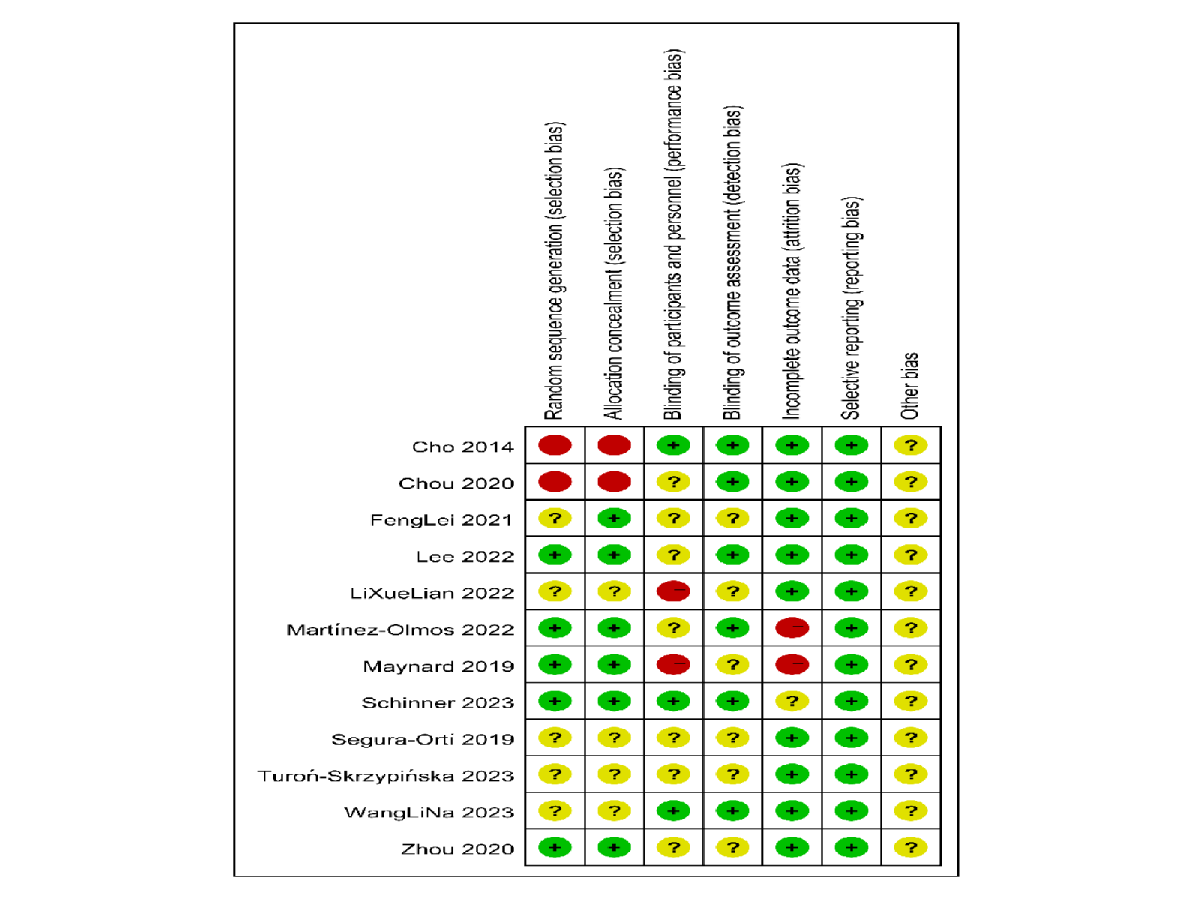
Risk of bias analysis of each included study [14-25].

According to Cochrane criteria, the risk of bias assessment is illustrated in Figures 2 [14-25] and 3. Of the 12 studies, 7/12 (58.3%) studies [14,15,17,18,21,22,24] were rated as having “some concerns” of bias, and 5/12 (41.7%) studies [16,19,20,23,25] were rated as high risk of bias. In the domain of “random sequence generation,” 5/12 (41.7%) studies [16,17,22,24,25] had low bias, 5/12 (41.7%) studies [14,15,18,20,21] had unclear risk of bias, and 2/12 (16.7%) studies [19,23] had high risk of bias. In the domain of “allocation concealment,” 6/12 (50%) studies [16-18,22,24,25] had low bias, 4/12 (33.3%) studies [14,15,20,21] had unclear risk of bias, and 2/12 (16.7%) studies [19,23] had high risk of bias. In the domain of “blinding of participants and personnel,” 3/12 (25%) studies [21,23,24] had low bias, 7/12 (58.3%) studies [14-19,22] had unclear risk of bias, and 2/12 (16.7%) studies [20,25] had high risk of bias. Blinding participants was not feasible due to the nature of the intervention methods used in all trials. In the domain of “blinding of outcome assessment,” 6/12 (50%) studies [16,19-21,23,24] had low bias and 6/12 (50%) studies [14,15,18,20,22,25] had unclear risk of bias. The absence of blinding outcome assessors or data analysts was observed in studies with some bias. In the domain of “incomplete outcome data,” 9/12 (75%) studies [14,15,17-23] had low bias, 1/12 (8.3%) studies [24] had unclear risk of bias, and 2/12 (16.7%) studies [16,25] had high risk of bias. In the domain of “selective reporting,” 12/12 (100%) studies [14-25] had a low bias.

**Figure 3:**
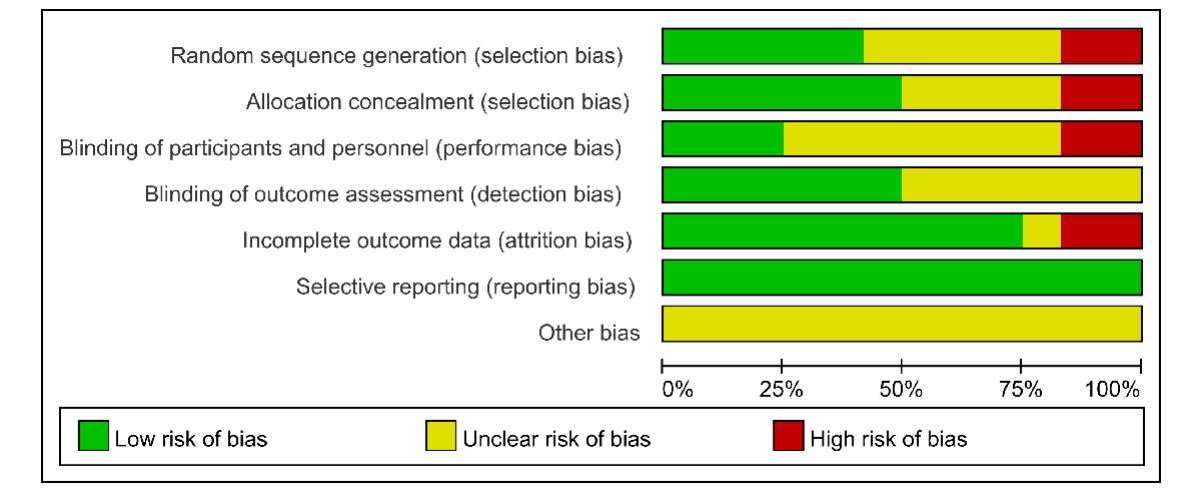
Overall risk of bias analysis of included studies.

### Meta-Analysis Results

#### Motor Ability

##### Gait Speed

**Figure 4:**
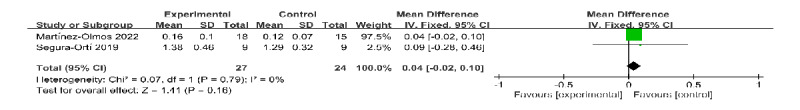
Forest plot of virtual reality on exercise capacity of gait speed [15,16].

Two trials used gait speed as a measurement outcome [15,16]. The results indicate no significant difference in gait speed improvement between VR training and other training methods (standardized mean difference [SD]=0.04, 95% CI –0.02 to 0.1, *P*=.16) with low heterogeneity (*I*^2^=0%, *P*=.79; Figure 4 [15,16]). Since there were fewer than 3 studies, publication bias was not assessed.

##### 6-Minute Walk Test

**Figure 5:**
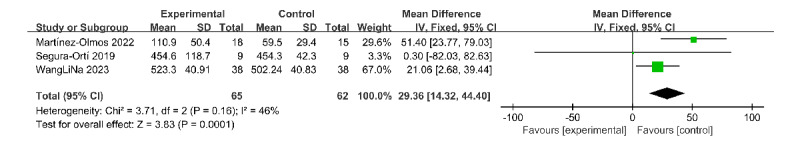
Forest plot of virtual reality on exercise capacity of 6-minute walk test [15,16,21].

**Figure 6:**
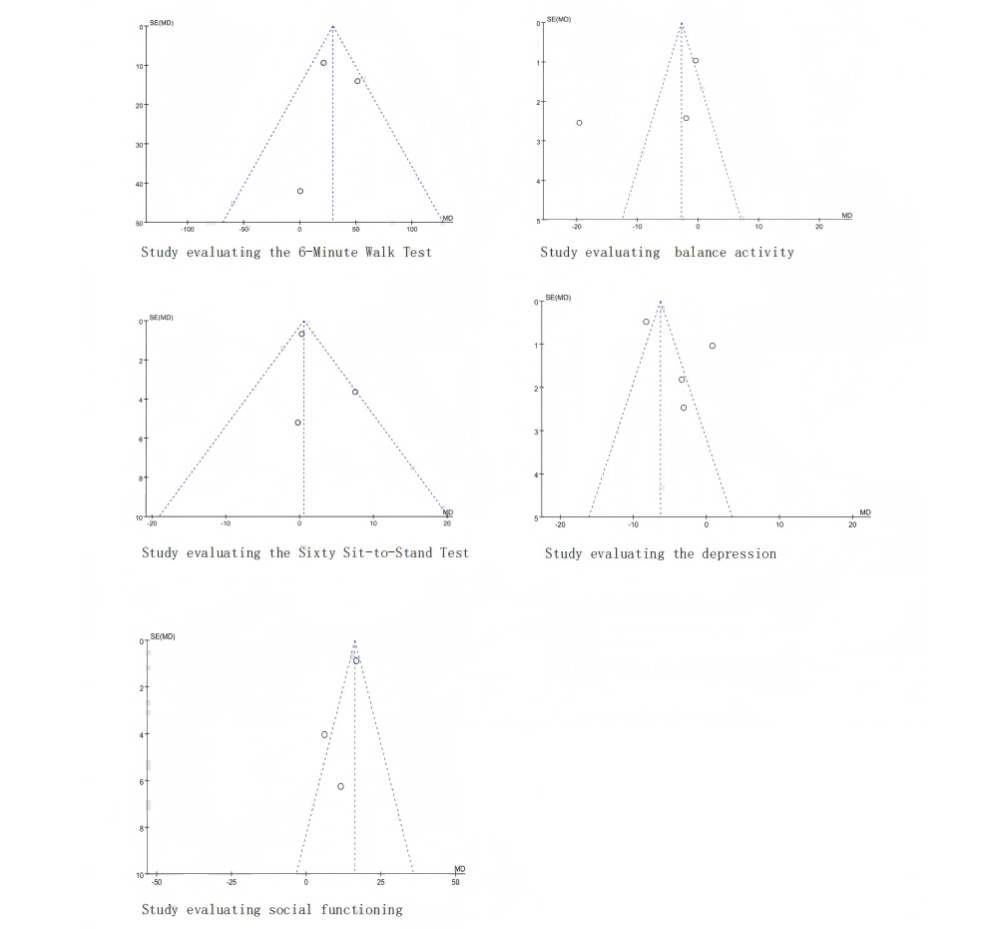
The funnel plot of the effect of virtual reality on dialysis patient.

Three trials measured walking ability using the 6-minute walk test (6MWT) [15,16,21]. Results showed that VR training improved walking ability significantly more than the control group (SD=29.36, 95% CI 14.32-44.4, *P*<.001) with middle heterogeneity (*I*^2^=46%, *P*=.16; Figure 5 [15,16,21]). Egger regression test and funnel plot showed that there was no significant publication bias between the included studies (*P*=.99; Figure 6).

##### Balance Ability

**Figure 7:**
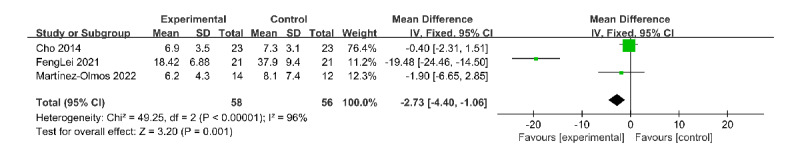
Forest plot of virtual reality on exercise capacity of balance [16,18,23].

Three trials included the measurement of balance ability as an outcome [16,18,23]. The findings suggest that VR training did not lead to an improvement in the balance ability of the subjects when compared with the control group (SD=–2.73, 95% CI –4.40 to –1.06, *P*<.001), and considerable heterogeneity was found (*I*^2^=96%, *P*<.001; Figure 7 [16,18,23]). Egger regression test and funnel plot showed that there was no significant publication bias between the included studies (*P*=.48; Figure 6).

##### Five Sit-to-Stand Test

**Figure 8:**
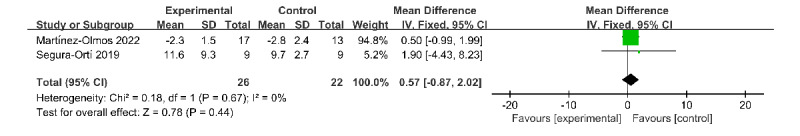
Forest plot of virtual reality on exercise capacity of Five Sit-to-Stand Test [15,16].

Two studies were conducted using the Sit-to-Stand Test 5 (STS-5) as a measurement outcome [15,16]. The results indicate that there was no significant difference in Sit-to-Stand Test performance between VR training and the control group (SD=0.57, 95% CI –0.87 to 2.02, *P*=.44) with low heterogeneity (*I*^2^=0%, *P*=.67; Figure 8 [15,16]). However, since there were fewer than three studies, publication bias was not assessed.

##### Ten Sit-to-Stand Test

**Figure 9:**
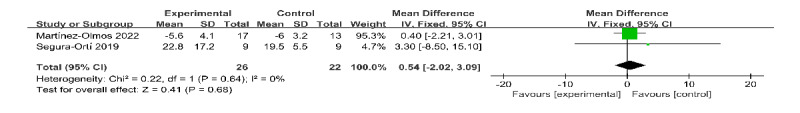
Forest plot of virtual reality on exercise capacity of Ten Sit-to-Stand Test [15,16].

In 2 trials [15,16], Sit-to-Stand Test 10 (STS-10) was used as a measurement outcome to compare the Sit-to-Stand Test performance between the VR training group and the control group. The results did not show any significant difference (SD=0.54, 95% CI –2.02 to 3.09, *P*=.68) with low heterogeneity (*I*^2^=0%, *P*=.64; Figure 9 [15,16]). As there were fewer than 3 studies, publication bias was not assessed.

##### Sixty Sit-to-Stand Test

**Figure 10:**
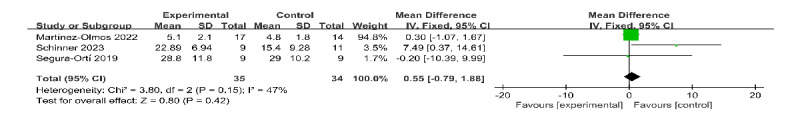
Forest plot of virtual reality on exercise capacity of Sixty Sit-to-Stand Test [16,18,24].

Three trials were conducted with the Sit-to-Stand Test 60 (STS-60) as the measurement outcome [15,16,24]. The results suggest that there was no significant difference in the STS-60 test performance between VR training and other training methods for dialysis patients (SD=0.55, 95% CI –0.79 to 1.88, *P*=.42) with middle heterogeneity (*I*^2^=47%, *P*=.15; Figure 10 [16,18,24]). Egger regression test and funnel plot showed that there was no significant publication bias between the included studies (*P*=.57; Figure 6).

##### Timed Up and Go Test

**Figure 11:**
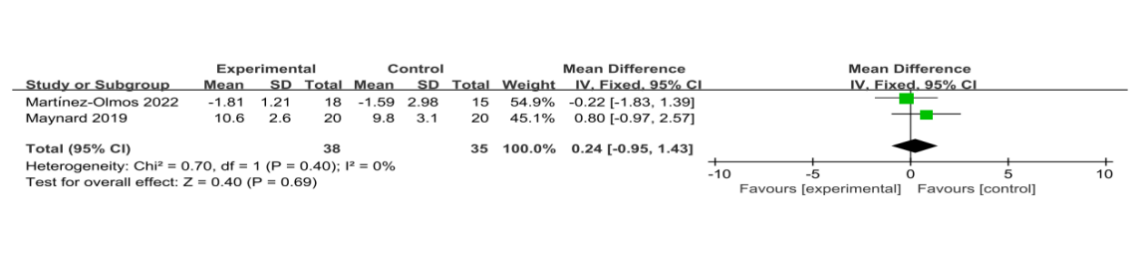
Forest plot of virtual reality on exercise capacity of Timed Up and Go Test [16,25].

Two trials included the Timed Up and Go Test (TUG) test as a measurement outcome [16,25]. The results showed that there was no significant difference between the VR training and control groups (SD=0.24, 95% CI –0.95 to 1.43, *P*=.69) with low heterogeneity (*I*^2^=0%, *P*=.40; Figure 11 [16,25]). However, since there were fewer than 3 studies, publication bias was not assessed.

#### Psychological Outcomes

##### Depression

**Figure 12:**
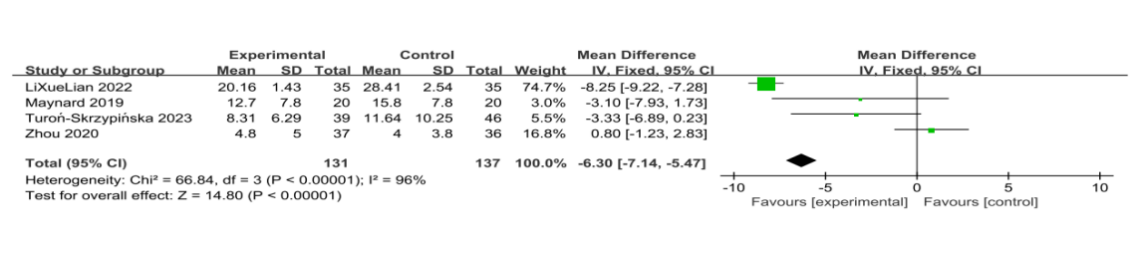
Forest plot of virtual reality on psychological outcome of depression [14,20,22,25].

Four trials used depression scores as outcome measures [14,20,22,25]. The results show that patients undergoing VR training had lower depression symptoms compared with the control group, indicating that VR was more effective in improving the participants’ depressive condition (SD=–6.30, 95% CI –7.14 to –5.47, *P*<.001) and considerable heterogeneity was found (*I*^2^=96%, *P*<.001; Figure 12 [14,20,22,25]). Egger regression test and funnel plot showed that there was no significant publication bias between the included studies (*P*=.34; Figure 6).

##### Anxiety

**Figure 13:**
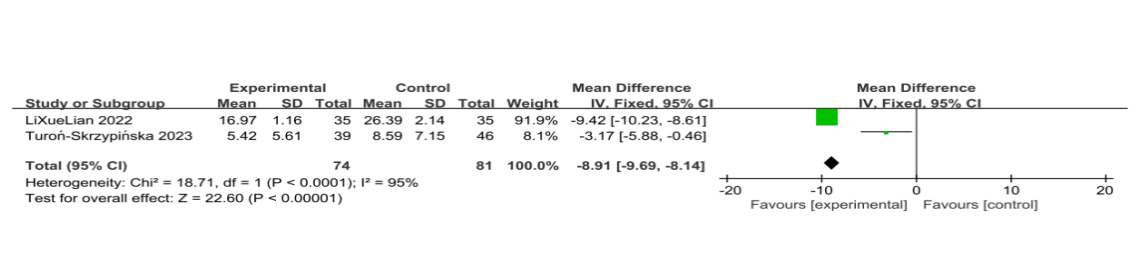
Forest plot of virtual reality on psychological outcome of anxiety [14,20].

Two trials used anxiety scores as outcome measures [14,20]. The results show that patients undergoing VR training had lower anxiety levels compared with the control group, indicating that VR was more effective in improving the participants’ anxiety condition (SD=–8.91, 95% CI –9.69 to –8.17, *P*<.001) with high heterogeneity (*I*^2^=95%, *P<*.001; Figure 13 [14,20]). Since there were fewer than 3 studies, publication bias was not assessed.

##### Fatigue

**Figure 14:**
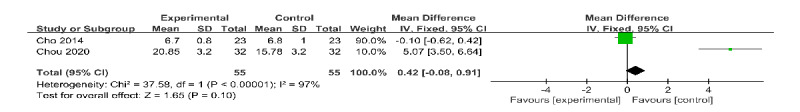
Forest plot of virtual reality on psychological outcome of fatigue [19,23].

Two trials used fatigue scores as outcome measures [19,23]. The results indicate no significant difference in improving fatigue among dialysis patients between VR training and other training methods (SD=0.42, 95% CI –0.08 to 0.91, *P*=.01) with high heterogeneity (*I*^2^=97%, *P*<.001; Figure 14 [19,23]). Since there were fewer than 3 studies, publication bias was not assessed.

#### Social Functioning

**Figure 15:**
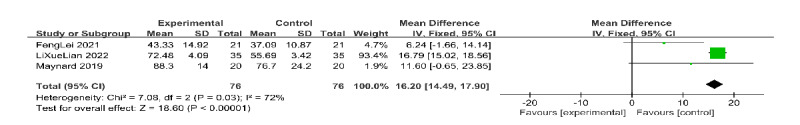
Forest plot of virtual reality on social function [18,20,25].

Three trials used social functioning scores as outcome measures [18,20,25]. The results demonstrate that VR training was more effective than the control group interventions in improving patients’ social functioning (SD=16.20, 95% CI 14.49-17.90, *P*<.001) with middle heterogeneity (*I*^2^=72%, *P*=0.03; Figure 15 [18,20,25]). Egger regression test and funnel plot showed that there was no significant publication bias between the included studies (*P*=.35; Figure 6).

#### Self-Efficacy

**Figure 16:**
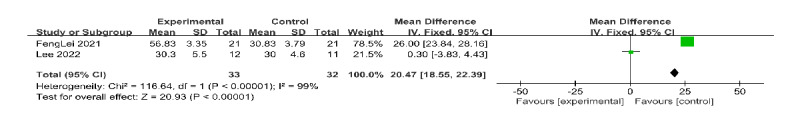
Forest plot of virtual reality on self-efficacy [17,18].

Two trials used self-efficacy scores as outcome measures [17,18]. The results indicate that VR training was more effective than the control group interventions in improving patients’ self-efficacy (SD=20.47, 95% CI 18.55-22.39, *P*<.001) with high heterogeneity (*I*^2^=99%, *P*<.001; Figure 16 [17,18]). Since there were fewer than 3 studies, publication bias was not assessed.

## Discussion

### Summary of Main Findings

This meta-analysis encompassed 12 trials involving 625 participants, of whom (99/625; 15.8%) were PD patients. All studies used VR training as the intervention, while control groups received conventional methods such as health education, routine care, and exercise adapted to patients’ daily routines. Of these 12 studies, 5 reported the use of random sequence generation.

This meta-analysis offers a thorough evaluation of the effectiveness of VR training in comparison with traditional methods across various outcome measures, including motor abilities, balance, and psychological well-being. The findings indicate that VR training significantly enhances walking ability, as measured by the 6MWT, with an SD of 29.36. This effect is notably more pronounced compared with gait speed and other motor ability measures, where VR training did not produce significant improvements. Furthermore, VR training was linked to significant reductions in symptoms of depression and anxiety, along with enhancements in social functioning and self-efficacy. However, VR training did not show significant benefits for balance, Sit-to-Stand tests, or the TUG.

### Physical Mobility

The substantial improvement observed in the 6MWT with VR training stands in stark contrast to the absence of significant results in gait speed. The 6MWT evaluates endurance and walking capability over a period, which may be well-suited to the interactive and engaging nature of VR environments. The immersive qualities of VR training may enhance patients’ motivation and engagement during prolonged walking tasks, thereby contributing to better performance on the 6MWT. Silva et al [26] demonstrated that visual and auditory stimuli in virtual environments encourage active participation in training, leading to increased exercise, walking distance, and time, which enhances the effectiveness of the 6MWT and improves patients’ walking ability. Research indicates that gait rehabilitation programs using VR can aid dialysis patients with muscle weakness and joint pain in improving walking speed and quality by simulating diverse walking environments and obstacles, thereby enhancing their independence in daily life [27]. The inclusion of dynamic, goal-oriented tasks in VR may better simulate real-world walking scenarios, motivating participants to exert greater effort and maintain activity for extended periods. In contrast, gait speed, a measure of immediate walking performance, may not benefit as substantially from the immersive features of VR training. The effectiveness of VR in enhancing gait speed may be influenced by the intensity and duration of training sessions, which varied across studies. Consequently, the lack of significant differences in gait speed improvement may suggest a need for more specific training protocols or supplementary interventions to optimize gait speed outcomes.

### Psychological Symptoms

The observed significant improvement in walking ability among dialysis patients underscores the potential of VR training to address key aspects of patient care. Improving walking capability can significantly impact daily functioning and overall quality of life, particularly in individuals with mobility impairments [28]. The capacity to enhance physical function while concurrently addressing psychological factors, such as depression and anxiety, highlights the comprehensive benefits of VR training. Research by Schröder et al [29] further demonstrates that VR training can alleviate patients’ negative emotions, including anxiety and depression. By offering a multifaceted therapeutic approach, VR has the potential to enhance both physical performance and mental well-being, essential components of holistic patient care. Furthermore, the effectiveness of VR in reducing depression and anxiety suggests that VR training may serve as a valuable tool for mitigating the broader psychosocial impacts of chronic illness [30]. The immersive and interactive features of VR provide an engaging and motivating environment that fosters positive changes in mood and self-perception, thereby contributing to improved overall patient outcomes [31].

### Social Functioning

The findings of this meta-analysis demonstrate that VR training significantly improves social functioning in patients, as evidenced by a SD of 16.2. This result suggests that VR training can substantially enhance social interaction and integration, which are crucial aspects of overall well-being. The positive effect of VR on social functioning may be attributed to various factors inherent to the VR experience. VR environments frequently offer engaging and interactive scenarios that promote social engagement and collaboration, even among individuals with limited mobility or social anxiety. VR training usually includes interactive tasks and group-based activities that encourage active participation, thereby enhancing patients’ social skills and interactions. The immersive nature of VR provides opportunities for patients to practice social scenarios in a controlled environment, thereby enhancing their confidence and comfort in real-world social interactions. Furthermore, participation in VR-based social activities may foster a sense of community and support, further enhancing social functioning. Despite these promising results, it is crucial to consider the variability in social functioning outcomes reported across studies. The observed heterogeneity (*I*²=72%) indicates that factors such as the design of VR interventions, the nature of social interactions, and the duration of training may affect the effectiveness of VR in enhancing social functioning. Future research should focus on exploring these variables in detail and identifying the specific components of VR training that most effectively improve social interaction and integration [32].

### Self-Efficacy

Virtual reality training demonstrated a significant improvement in self-efficacy, with an SD of 20.47, indicating that VR interventions substantially enhance patients’ confidence in their ability to manage various tasks and challenges. Self-efficacy, defined as the belief in one’s ability to achieve goals, is a crucial determinant of both physical and psychological health. The observed improvement in self-efficacy with VR training can be attributed to the interactive and engaging nature of VR environments, which frequently include goal-setting, feedback, and achievement [33]. VR training typically integrates elements such as progress tracking, rewards, and virtual challenges, which can enhance patients’ confidence in their abilities. By offering immediate feedback and a sense of accomplishment, VR can reinforce positive behaviors and attitudes, thereby contributing to improved self-efficacy. Furthermore, the immersive and supportive features of VR environments may help patients overcome fears and build confidence in a controlled setting, potentially translating into improved self-efficacy in real-world situations [34]. However, the high heterogeneity (*I*²=99%) in the self-efficacy outcomes suggests that the effectiveness of VR in enhancing self-efficacy may be influenced by several factors, including the design of the VR interventions and individual patient characteristics. Future research should focus on identifying the most effective VR components for enhancing self-efficacy and investigating how different patient characteristics may influence the outcomes of VR training.

### Challenges and Limited Use of VR in PD Patients

The use of VR training among PD patients has been relatively limited compared with other patient populations. Several factors are likely to contribute to this limited use. PD patients face distinct challenges, such as the requirement for specialized equipment and potential complications related to fluid management, which may complicate the integration of VR training. In addition, this patient population may have unique physical and psychological conditions that necessitate tailored interventions. Current research on VR training for this population is still emerging, and the application of VR may need adaptation to address these specific needs effectively.

Furthermore, PD patients may experience varying levels of physical capability and psychological stress compared with other patient groups, which may influence the outcomes of VR interventions. Future research should focus on developing and testing VR programs specifically tailored for this population, considering their unique requirements and limitations to ensure that the interventions are both safe and effective.

### Advantages and Limitations

This meta-analysis highlights the potential of VR training as an adjunctive therapy for enhancing walking ability and psychological well-being in dialysis patients. The substantial improvements in walking endurance and psychological outcomes observed in this analysis suggest that VR training may be a valuable addition to existing therapeutic strategies. Nevertheless, the observed variability in results and heterogeneity in certain outcomes underscores the need for further research.

The strengths of this study include (1) it is the first meta-analysis to assess the impact of VR training on dialysis patients and (2) we assessed the effectiveness of VR training using 12 outcome measures: gait speed, balance, 6-minute walk test score, STS-5, STS-10, STS-60, TUG, depression, anxiety, fatigue, social functioning, and self-efficacy, providing a comprehensive reference for future research.

This study also has limitations: (1) it included only published articles in Chinese and English, which may impact the meta-analysis results, (2) some studies did not provide details on sequence generation, allocation concealment, and blinding, and (3) the 12 studies included in the analysis used different control interventions and the intervention durations across the studies varied significantly, which may have contributed to significant heterogeneity among the studies.

Future research should focus on refining VR interventions, exploring their optimal implementation, and addressing the specific needs of diverse patient populations. Larger and more diverse sample sizes, coupled with standardized training protocols, will be crucial for comprehensively evaluating the benefits and limitations of VR.

In this study, we used multiple databases for literature retrieval to ensure comprehensive coverage of relevant research in the field. Our selected databases included CNKI, Wanfang, China Science and Technology Journal Database, SinoMed, Cochrane Library, Web of Science, PubMed, and Embase. These databases offer a wealth of research outputs, particularly the Chinese databases, which aggregate a substantial amount of high-quality studies conducted by Chinese scholars, reflecting the region’s active contribution to this field. However, we recognize that our current search strategy may not fully represent the entire research landscape, particularly the lack of studies from North America.

Although our initial search did not yield relevant North American research, this may be attributed to the limitations of our search strategy and keyword selection. We acknowledge that this omission could impact the representativeness of our results and potentially influence the conclusions drawn from the study. Therefore, we emphasize this limitation within the article and recommend that future research consider broader literature retrieval strategies to ensure a more holistic understanding of the field.

This reflection provides important guidance for subsequent studies, ensuring that future efforts can better integrate research findings from diverse regions, thereby enhancing the breadth and depth of analysis.

### Conclusions

This meta-analysis offers a thorough evaluation of the use of VR training in dialysis patients. The findings indicate that VR training effectively enhances patients’ physical fitness, alleviates anxiety and depression, and improves adherence, thereby facilitating better self-management. However, research focusing on VR training in peritoneal patients is relatively limited compared with that on hemodialysis patients. Future research should involve PD patients and use rigorous, large-scale studies to validate the findings of this review for both hemodialysis and PD populations.

## Appendix

Multimedia Appendix 1 PRISMA (Preferred Reporting Items for Systematic Reviews and Meta-Analyses) checklist.

Multimedia Appendix 2 Search strategies of PubMed, the Cochrane Library, the China National Knowledge Infrastructure (CNKI) and the VIP Journal Database. Supplementary information of intervention in experimental and control groups.

Multimedia Appendix 3 Summary of included studies.

## Abbreviations

6MWT: 6-minute walk test
CNKI: China National Knowledge Infrastructure
ESRD: end-stage renal disease
HD: hemodialysis
PD: peritoneal dialysis
PRISMA: Preferred Reporting Items for Systematic Reviews and Meta-Analyses
SD: standardized mean difference
STS-10: Sit-to-Stand Test 10
STS-5: Sit-to-Stand Test 5
STS-60: Sit-to-Stand Test 60
TUG: Timed Up and Go Test
VR: virtual reality
VRT: virtual reality training

## Data Availability

All data generated or analyzed during this study are included in this published article and Multimedia Appendices 1-3.
