# Supplementary material for: Effectiveness of Virtual Reality Training in Improving Outcomes for Dialysis Patients: Systematic Review and Meta-Analysis
Source: J Med Internet Res. 2025 Jan 8;27:e58384. doi: 10.2196/58384 (PMC11754980; doi:10.2196/58384)
Supplement: Multimedia Appendix 2 [file jmir_v27i1e58384_app2.doc]

## Multimedia Appendix 2

## Search strategies

Cochrane Library（34）

#1 Virtual Reality

#2 Augmented reality

#3 User-Computer Interface

#4 Video Games

#5 Video game* OR virtual reality exposure therapy OR sensor OR exergame OR digital health OR “Nintendo Wii，Kinect” OR Xbox

#6 #1 OR #2 OR #3 OR #4 OR #5

#7 Renal dialysis

#8 Dialysis

#9Peritoneal Dialysis

#10H$modialysis OR Extracorporeal Dialys* OR hemodialys* OR renal dialys* OR hemofiltration OR hemodiafiltration

#11 #7 OR #8 OR #9 OR #10

#12 #6 AND #11

PubMed（656）

("video game*"[Title/Abstract] OR "virtual reality exposure therapy" OR sensor OR exergame OR digital health OR "Nintendo Wii，Kinect" OR Xbox OR "Virtual Reality"[Mesh] OR "Augmented Reality"[Mesh] OR "User-Computer Interface"[Mesh] OR "Video Games"[Mesh]) AND (H$modialysis OR "Extracorporeal Dialys*" OR hemodialys* OR "renal dialys*" OR hemofiltration OR hemodiafiltration OR "Renal Dialysis"[Mesh] OR "Dialysis"[Mesh] OR"Peritoneal Dialysis"[Mesh])

中国知网数据库（80）

（主题：虚拟现实 + 虚拟现实暴露疗法 + 虚拟暴露疗法 + 虚拟 + 虚拟游戏 + 电脑虚拟活动 + 电脑虚拟活动 + 互动电子游戏）AND（主题：肾透析 + 血液透析 + 腹膜透析 + 透析 + 体外透析 + 血液透析滤过）

维普数据库（121）

((((((任意字段=肾透析 OR 任意字段=血液透析) OR 任意字段=腹膜透析) OR 任意字段=透析) OR 任意字段=体外透析) OR 任意字段=血液透析滤过) AND (((((((任意字段=虚拟现实 OR 任意字段=虚拟现实暴露疗法) OR 任意字段=虚拟暴露疗法) OR 任意字段=虚拟) OR 任意字段=虚拟游戏) OR 任意字段=电脑虚拟活动) OR 任意字段=电脑虚拟活动) OR 任意字段=互动电子游戏)) AND (years:[1900 TO 2023])
