# Supplementary material for: Effectiveness of Virtual Reality Training in Improving Outcomes for Dialysis Patients: Systematic Review and Meta-Analysis
Source: J Med Internet Res. 2025 Jan 8;27:e58384. doi: 10.2196/58384 (PMC11754980; doi:10.2196/58384)
Supplement: Multimedia Appendix 3 [file jmir_v27i1e58384_app3.docx]

## Multimedia Appendix 3

Table 1 Characteristics of the 12 included studies

| Author  （year） | Country | Type | Number | | | Duration | Time of data collection | Outcomes |
| --- | --- | --- | --- | --- | --- | --- | --- | --- |
|  |  |  | Total | Experimental group | Control group |  |  |  |
| Turoń-Skrzypińska et al  (2023) | Poland | HD | 85 | 39 | 46 | 1 year | 1.at the time of entering the research project  2.following month three | 1.depression  2.anxiety |
| Lee et al  (2022) | China | PD | 23 | 12 | 11 | 8 times | 1.at baseline (pre-treatment), 2.within the first week after recruitment  3.at post-treatment | 1.knowledge proficiency  2.procedure competence 3.self-efficacy |
| Segura‐Ortí et al  (2019) | Spain | HD | 18 | 9 | 9 | 20 weeks | 1.at baseline 2.after 16 weeks of intradialysis combined exercise  3.by the end of four additional weeks of exercise | 1.STS-10^a^, STS-60^b^  2.gait speed, one-leg heel-rise tests  3.6MWT^c^ |
| Martínez-Olmos et al  (2022) | Spain | HD | 56 | 28 | 28 | 12 weeks | 1.at baseline  2.at 12 weeks  3.at 24 weeks | 1.the 4-m gait speed test  2.SPPB^d^  3.TUG^e^ test  4.OLST^f^ for balance  5. STS-10  6.STS-60  7.6MWT  8.adherence to the exercise programme |
| Feng Lei et al  (2021) | China | HD | 42 | 21 | 21 | 6 mouths | 1.Before enrollment  2.at 3 months  3.6 months after enrollment | 1.self-efficacy for exercise  2.risk of falls  3.quality of life |
| Zhou et al  (2020) | Qatar | HD | 73 | 37 | 36 | 4 weeks | 1.at baseline  2.at 4 weeks | 1.depression  2.user experience |
| Chou et al  (2020) | China | HD | 64 | 32 | 32 | 4 weeks | 1.Before intervention  2.after 4 weeks of intervention | 1.fatigue  2.BUN^g^  3.creatinine  4.albumin  5.hemoglobin |
| Cho et al  (2014) | South Korea | HD | 46 | 23 | 23 | 8 weeks | 1.at baseline  2.after intervention | 1.physical fitness  2.body composition  3.fatigue |
| Schinner et al  (2023) | Germany | HD | 32 | 12+9 | 11 | 12 weeks |  | 1.functional capacity  2.serum biochemistry  3.muscle strength  4.muscle circumference  5.body composition |
| Maynard et al  (2018) | Brazil | HD | 40 | 20 | 20 | 12 weeks | after intervention | 1.functional capacity  2.quality of life  3.depressive symptoms |
| Li Xuelian et al  (2022) | China | HD | 70 | 35 | 35 |  | 1.before intervention  2.after intervention | 1. anxiety  2. depression  3. quality of life  4.treatment compliance |
| Wang Lina et al  (2023) | China | PD | 76 | 38 | 38 | 3 mouths |  | 1.activity level  2.6MWT |

a.sit-to-stand 10, b.sit-to-stand 60, c.6-min walking test, d.Short Physical Performance Battery e.timed up-and-go, f.one-legged stance test, g.Blood urea nitrogen

Table 2. Supplementary information of intervention in experimental and control groups

| Studies | Experimental group | Control group |
| --- | --- | --- |
| Turoń-Skrzypińska et al [14] | The study group comprised patients undergoing hemodialysis as renal replacement therapy. Participants engaged in VR^a^ exercises using the prototype NefroVR system for 20 minutes during their hemodialysis sessions. These exercises were conducted 3 times a week within the first 1-2 hours of hemodialysis treatment or until achieving an ultrafiltration of 2.5 liters. | The control group consisted of patients undergoing renal replacement therapy by hemodialysis who were not assigned a task. |
| Lee et al [17] | In addition to standard IPD^b^, participants underwent a minimum of 8 additional sessions of VR^a^ training during PD^c^ exchanges. | Participants received educational materials on managing CAPD^d^ during their initial IPD^b^ session. This included information on disease understanding, different types of renal replacement therapy, multidisciplinary support, and common patient issues. |
| Segur-Ortí et al [15] | Participants in this group underwent a 16-week intradialytic combined exercise program consisting of aerobic and strengthening exercises, followed by an additional 4 weeks of intradialytic VR^a^ gaming. Each session began with a 5-minute warm-up, after which participants engaged in VR^a^ activities for up to 30 minutes, adjusting the duration based on their rate of perceived exertion, targeted between “somewhat hard” to “hard”. Exercise intensity progressed by increasing the number of 3-minute exercise bouts, ranging from 1 to 10, with 1 minute of rest between bouts. | Participants underwent a 16-week intradialytic combined exercise program consisting of aerobic and strengthening exercises, followed by an additional 4 weeks of intradialytic combined exercise program. The program included both strengthening and aerobic exercises. Each session began with a 5-minute warm-up, followed by strengthening exercises, basic equipments were used for these exercises. Participants then engaged in aerobic training using a cycloergometer for up to 30 minutes, concluding with a 5-minute stretching period. |
| Martínez-Olmos et al [16] | During the 12-week exercise period, participants engaged in three VR^a^ sessions per week coinciding with their dialysis treatment, conducted within the first 2 hours of the session with standard monitoring of blood pressure and heart rate. Each session began and ended with a 5-minute warm-up and cool-down, involving free hip, knee, and ankle flexion and extension movements. VR^a^ sessions lasted up to 40 minutes, adjusted based on participants’ perceived exertion, ideally falling between “somewhat hard” to “hard” (12-15 on the RPE^e^ scale). Exercise intensity progressed by increasing the number of exercise sets (each lasting 3-6 minutes), ranging from 1 to 6 sets with 1-minute rest intervals. | This study used a randomized, crossover, controlled trial design. Eligible participants were randomly assigned to receive either the VR^a^ intervention for 12 weeks followed by the control treatment for another 12 weeks. |
